# Microleakage of Class V Methacrylate and Silorane-based Composites and Nano-ionomer Restorations in Fluorosed Teeth

**Published:** 2015-06

**Authors:** Fereshteh Shafiei, Mohadese Abouheydari

**Affiliations:** 1 Biomaterial Research Center, Dept. of Operative Dentistry, School of Dentistry, Shiraz University of Medical Sciences, Shiraz, Iran;; 2 Postgraduate Student, Dept. of Operative Dentistry, School of Dentistry, Shiraz University of Medical Sciences, Shiraz, Iran;

**Keywords:** Microleakage, Methacrylate Composite, Silorane Composite, Nano-ionomer, Fluorosed Teeth

## Abstract

**Statement of the Problem:**

Enamel and dentin marginal sealing ability of the new adhesive materials could play an important role in successful restoration on fluorosed teeth.

**Purpose:**

The aim of this *in vitro* study was to evaluate the marginal microleakage of low-shrinkage silorane-based composite, nano-ionomer, and methacrylate-based composite through self-etching approach or with enamel acid etching.

**Materials and Method:**

Seventy-two extracted human molars with moderate fluorosed (according to Thylstrup and Fejerskov index, TFI= 4-6) were randomly divided into six groups (n=12). Class V cavities were prepared on the buccal surface at the cementoenamel junction and restored with Clearfil SE Bond/Clearfil AP-X (methacrylate composite), Silorane Adhesive System/Filtek P90 , and nano primer/nano-ionomer according to the manufacturer’s instructions (self-etching approach) or with additional selective enamel acid etching before primer application for each adhesive. After water storage and thermocycling, microleakages of the samples were assessed using dye-penetration technique at the enamel and dentin margins. Data were analyzed using non-parametric tests (α = 0.05).

**Results:**

There was a significant difference among the six groups at the enamel margin (*p*= 0.001), but not at the dentin margin (*p*= 0.7). For all the three adhesive materials, additional enamel etching resulted in significantly reduced microleakage at the enamel margin (*p*< 0.05).

**Conclusion:**

Methacrylate- and silorane-based composites and nano-ionomer revealed a similar and good performance in terms of dentin marginal sealing, but not at the enamel margin. The additional selective enamel etching might improve enamel sealing for the three materials.

## Introduction

Although role of fluoride in caries prevention is well-established, fluorosis is a side effect of its excessive intake.[1-2] Dental fluorosis is a kind of tooth malformation due to systemic overexposure to fluoride during tooth development. Drinking water containing high levels of fluoride, fluoride-containing supplements and dietary products can be sources of fluoride.[1-2]

The relatively high prevalence of fluorosis has been reported in different regions of Iran such as 100% in Makoo, 67% in Larestan and Bandar Lengeh and 67-80% in Dayer.[3-4]

Fluorotic enamel reveals two layers: an acid resistant surface layer (hypermineralized with fluorapatite) and a porous hypomineralized subsurface layer.[2] Fluorosis severity has been classified based on the Thylstrup and Fejerskov-index (score 0-9 for normal, mild, moderate, and severe fluorosis). The clinical appearance of fluorosis is correlated to the histopathologic changes in the enamel by this index.[5]

It has been found that with increasing fluorosis severity, the more porous subsurface enamel extends toward the inner enamel. These histological changes might result in chipping away the rather brittle well-mineralized surface enamel.[6]The subsequent exposure of the subsurface layer to surface attrition may lead to dentin exposure. Covering the dentin with an efficient adhesive material is necessary. In contrast to the moderately fluorosed enamel which is caries resistant, mild and moderately fluorosed dentin is shown to be caries susceptible.[7]This may be a result of changed morphology of the moderately fluorosed dentin, exhibiting hypomineralized areas of interglobular dentin with unfused minerals.[8]

On the other hand, adhesive bonding to the fluorosed enamel has been demonstrated to be problematic due to further resistance to acid dissolution of fluorapatite than hydroxyapatite.[5] Therefore, establishment of effective adhesive bonding to both fluorosed enamel and dentin is of major importance for a successful adhesive restoration in the fluorosed teeth.

Some authors have recommended that the hypermineralized layer be removed by grinding away the outer surface using a diamond bur.[9] However, in some clinical situations, unground fluorosed enamel might be involved in adhesive restorations such as resin composite placement over the cavity margins without bevel.[9-10] Preservation of caries resistant enamel margin could be beneficial for durable restoration of the fluorosed teeth. Few studies reported bond strength of some adhesives to unground fluorosed enamel and dentin.[9, 11-12] Only one study evaluated microleakage of Class V composite restoration on mid-buccal/lingual surfaces of fluorosed teeth and reported a higher leakage in self-etched teeth than total-etched ones.[13]

Therefore, this study was designed to investigate dentin and enamel marginal sealing of Class V cavities restored by using low-shrinkage resin materials, nano-ionomer (NI) and silorane-based composite compared with methacrylate-based composite associated to a two-step self-etch adhesive. Also, the effect of an additional acid etching of enamel margin along with the three adhesive materials on marginal sealing was evaluated in fluorosed teeth. 

## Materials and Method

In this experimental study, 72 caries-free extracted human molar teeth were collected from 20-35 year old patients, living in fluorosis endemic areas of Iran with moderate fluorosis (TFI= 4 to 6).[5] The teeth were cleaned and stored in a 0.1% thymol solution during the three months taken for accumulation of the teeth. Standard Class V cavities (5mm wide, 3 mm high, and 2 mm deep) with the gingival margin of 1mm below the cementoenamel junction were prepared on the buccal surface of the teeth using fissure diamond burs (Teezkavan; Tehran, Iran) in an air/water cooled high speed turbine. The bur was replaced for every five preparations. The prepared teeth were randomly divided into six groups (n=12) according to the used adhesive procedures/ materials.

Group 1 (SEB/Methacrylate C): Primer of Clearfil SE Bond (SEB; Kuraray, Okayama, Japan) was applied on the cavity surface for 20 seconds and then gently air dried. The bond was applied; gently air dried and light cured for 10 seconds. The cavity was restored with methacrylate composite, Clearfil AP-X (Kuraray; Okayama, Japan) in two increments; each was light-cured for 20 seconds. 

Group 2 (Etch+SEB/Methacrylate C): The enamel margin of the cavities was etched with 37% phosphoric acid (Denfil; Vericom Co., Korea) for 30 seconds, washed for 30 seconds and dried. Clearfil SE Bond was then applied and cavity was restored as described in group 1. 

Group 3 (SAS/Silorane C): Primer of Silorane Adhesive System (SAS, 3M ESPE; St. Paul, MN, USA) was applied for 10 seconds, gently air dried, and light cured for 10 seconds. Bond was applied gently, air thinned, and light cured for 10 seconds. The cavity was restored with silorane-based composite (Filtek P90, 3M ESPE; St. Paul, MN, USA) in two increments, each light cured for 40 seconds. 

Group 4 (E+SAS/Silorane C): After acid etching, washing and drying the enamel margin, SAS was applied and filling was done similar to group 3.

Group 5 (Nano-primer/NI): Nano-primer (3M ESPE) was applied for 15 seconds; air dried, and light cured for 10 seconds. Two parts of nano-ionomer (NI; Ketac N100, 3M ESPE) were mixed and placed into the cavity. The restoration was cured for 40 seconds. 

Group 6 (E+Nano primer/NI): All restorative procedures were the same as what was described for all other groups, except for the prior enamel acid etching for 30 seconds. 

All curing steps were done using a light-curing unit (VIP Junior; Bisco, Schaumburg, IL, USA) at 650 mW/cm[2]light intensity. After water storage for 24 h, all restored teeth underwent 1000 thermal cycles between 5-55C^o^ in water baths (TC-300; Vafaei Industrial, Tehran, Iran) with 30-second dwell time. The root apices of the teeth were then sealed with utility wax, and all the surfaces except for the fillings and 1 mm from the margins, were covered with two layers of nail polish. They were immersed in a 0.5% methylene blue solution for 24 hours. The teeth were then washed with water, blot-dried and sectioned through the center of the fillings faciolingually with a water-cooled diamond wheel saw (Leitz 1600; Wetzlar, Germany). Dye penetration in the sections was assessed in a blinded manner by two evaluators using a stereomicroscope (Carl Zeiss Inc.; Oberkochen, Germany) at ´20 magnifications.

The dye penetration extents were scored for both the enamel and dentin margins from 0-3 as follows: 0= no dye penetration; 1= penetration of dye along the cavity wall, but less than one half of the length; 2= penetration of dye along the cavity wall, but short of the axial wall; 3= penetration of the dye to and along the axial wall.[14]

The obtained results were statistically analyzed using Kruskal-Wallis and Mann-Whitney U non-parametric tests at *p*< 0.05 level of significance.

## Results

Microleakage scores for the enamel and dentin margins of the six groups are presented in Table 1. Kruskal-Wallis test indicated a significant difference among the six groups at the enamel margin (*p*= 0.001), but not at the dentin margin (*p*= 0.07).

**Table 1:** Microleakage scores obtained from the six groups

**Score** **Groups**	**Enamel Margin**					**Dentin Margin**				
	**0**	**1**	**2**	**3**	**Median**	**0**	**1**	**2**	**3**	**Median**
1) SEB/Methacrylate C	5	1	2	4	1.50	7	3	2	0	0.0
2)Etch+SEB/Methacrylate C	9	2	1	0	0.0	6	2	3	1	0.5
3)SAS/Silorane C	3	5	1	3	1.0	8	3	1	0	0.0
4) Etch+SAS/Silorane C	10	1	1	0	0.0	7	1	3	1	0.0
5) Nano primer/NI	3	2	4	3	2.0	8	4	0	0	0.0
6) Etch+Nano primer/NI	10	2	0	0	0.0	7	2	2	1	0.0

SEB: Clearfil SE Bond                   C: Composite resin      Etch: Acid etching       SAS: Silorane Adhesive System       NI: Nono-ionomer

Pairwise multiple comparisons of each adhesive material (with and without enamel etching) at the enamel margin was performed using Mann-Whitney U-test, which showed a significant difference between groups 1 and 2 (*p*= 0.04), 3 and 4 (*p*= 0.006), and 5 and 6 (*p*= 0.002) (Table 2). These results demonstrated the beneficial effect of enamel etching on sealing ability of the three adhesive materials used at the enamel margin.

There was no significant difference between groups 1 and 3, 1 and 5, 3 and 5, 2 and 4, 2 and 6, and 4 and 6 (*p*> 0.05) (Table 2), revealing a similar leakage for the three adhesive materials in the case of no etching and when etching the enamel margin was performed.

**Table 2:** Pairwise comparisons of the six groups at the enamel margin

	**G1**	**G2**	**G3**	**G4**	**G5**	**G6**
G1		*p*= 0.04	*p*= 1	*p*= 0.02	*p*= 0.7	*p*= 0.01
G2	*p*= 0.04		*p*= 0.01	*p*= 0.6	*p*= 0.007	*p*= 0.5
G3	*p*= 1	*p*= 0.01		*p*= 0.006	*p*= 0.5	*p*= 0.003
G4	*p*= 0.02	*p*= 0.6	*p*= 0.006		*p*= 0.004	*p*= 0.9
G5	*p*= 0.7	*p*= 0.007	*p*= 0.7	*p*= 0.004		*p*= 0.002
G6	*p*= 0.01	*p*= 0.5	*p*= 0.003	*p*= 0.9	*p*= 0.002	

*p< 0.05 is considered significant

## Discussion

With increasing prevalence of fluorosis in many areas of the world and widespread use of resin materials for adhesive restoration of the fluorosed teeth,[15] achieving durable marginal sealing is a concern. This sealing is capable of preventing microleakage, recurrent carries and pulpal pathology. Polymerization shrinkage of resin materials and the resultant stresses on early developing of bonding interface can lead to gap formation at margins of restorations. This occurrence could be minimized by reducing polymerization shrinkage and increasing quality of the adhesive bond.[16]

New silorane containing resin monomers from combination of siloxane and oxirane have been developed based on cationic ring opening polymerization, resulting in reduced polymerization shrinkage of silorane-based composite.[17]

Nano-ionomer (NI) is a novel highly packed nanofilled resin-modified glass-ionomer that has been recently introduced to dental market. In addition to advantages of RMGI, NI showed improved mechanical strength, resistance to biomechanical degradation and lower polymerization shrinkage.[18-20] These two types of resin materials might be a desirable restoration for fluorosed teeth. Ermis *et al.*[10]recommended that a good two-step self-etch adhesive along with selective enamel acid etching could provide reliable bonding to fluorosed teeth. Waidyasekera *et al.*[12] reported that the two-step self-etch adhesive, SEB revealed a higher bonding performance to fluorosed dentin than etch-and-rinse and one-step self-etch adhesives. The separate hydrophobic bonding resin could provide better dentinal sealing.[10]

In the current study, all adhesive materials showed a similar slight microleakage the dentin margin. Silorane composite was used associated with a two-step self-etch adhesive similar to Clearfil SE Bond. Silorane Adhesive System consists of a hydrophilic ultra-mild self-etch (pH=2.7) primer and a hydrophobic bond which was separately applied and light-cured. The resin layer has demonstrated to maintain the normal dentin-adhesive interface sealed against the ingress of water.[21]

The incompletely mineralized fluorosed dentin with inhomogeneous mineral distribution has more water permeability.[8, 12, 22] The separate hydrophobic resin layer of two-step self-etch adhesives used, particularly the bond component of Silorane Adhesive System, could seal the dentin; this type of resin sealing might contribute to an adequate dentinal sealing observed in this study. Ermis *et al.* concluded that moderate fluorosis does not influence the bond strength of SEB to dentin.[23]

On the other hand, higher acid susceptibility of fluorosed dentin[7] resulted in more aggressiveness of separate phosphoric acid etching on the dentin.[12] Therefore, in the current study, the dentin was not etched; the mild and ultra-mild self-etching primers of self-etch adhesives used might bond to fluorosed dentin better than etch-and-rinse adhesives do.

NI bonds to the tooth structures using a self-etching nano primer (pH=3). This light-cured primer contains a monomer and a photoinitiator that may create a resin covering on the primer dentin similar to those of mild one-step self-etch adhesive.[24] It seems that water permeation through the nano-primed fluorosed dentin may not only have no adverse effect on NI, but also may provide sufficient water for the maturation of NI as a glass-ionomer based material. These might explain the excellent dentinal sealing obtained in NI restoration of fluorosed teeth.

Considering low etching efficacy of self-etch adhesives and their questionable bonding ability to the enamel,[25] some authors recommended selective enamel etching prior to the adhesives.[26-27] The unreliable enamel bonding might be relevant to nano-primer/NI due to insufficient acidity of nano-primer, especially on the hypermineralized fluorosed enamel. A one-year clinical evaluation of NI restorations in normal teeth disclosed enamel marginal deficiencies.[24] This explanation may account for the higher enamel leakage observed in the nano-primer/NI group than enamel acid-etching plus nano primer group. According to Ermis *et al.*,[9] bonding effectiveness of mild self-etch adhesive to unground fluorosed enamel was lower than the etch-and-rinse adhesive. The latter showed no significant difference in bond strength between fluorosed and normal enamel. Also, Ertugrul *et al.*[11] confirmed stronger bonding of etch-and-rinse adhesives to unground fluorotic enamel compared with those of self-etch adhesives. In the two cited studies,[9, 11] enamel acid was etched for 15 seconds for normal and fluorosed enamels. In accordance with these reports, acid etching of the enamel margins for 30 seconds prior to the two self-etch adhesives used improved marginal sealing. This procedure had no effect on dentinal sealing. It seems that strengthening the enamel adhesion through acid-etching may not have any effect on the adequate dentin adhesion of the three adhesive materials used in this study.

The lower microleakage in total-etched teeth for 60 seconds as compared with 30 seconds and a higher leakage in self-etched teeth than total-etched teeth were reported for moderate fluorosed teeth with the cavity surrounding the enamel.[12] Although some authors recommended long etching time (90 seconds),[15] surface roughness and depth profile analyses indicated that 30 seconds etching time provide the best results for moderate fluorosed enamel.[28] This beneficial effect of enamel etching could be attributed to acid resistance of outer hypermineralized layer of moderate fluorotic enamel. The preservation of this layer might contribute to higher resistance of the fluorosed teeth to further deterioration.[28] In contrast to our findings, it was reported that moderate fluorosis had no adverse effect on enamel bonding ability of moderate and mild self-etch adhesives.[29]

The specific tooth type and the age group used in this study minimized the effect of these factors on the fluoride content and consequent enamel adhesion.[11, 30] The used teeth classified as TFI score 4-6 (moderate fluorosis) exhibited chalky white appearance and distinct pitting area on the enamel surface. In higher TFI scores, considerable parts of the surface enamel are lost.[5] Moreover, the fluoride level of the surface enamel in fluorosed teeth with TFI=7-8 was reported to be similar to that in the teeth with TFI=5-6.[31] Further studies are required to evaluate the interaction of new adhesive materials with tooth structures involved in different severity of fluorosis.

## Conclusion

According to our results, it can be concluded that the three adhesive materials based on self-etching approach, nano-primer/NI, Silorane Adhesive/Silorane composite and SEB/methacrylate composite revealed a good performance at the dentin margin but a poor performance at the enamel margin in terms of marginal sealing. Selective enamel acid etching improved the enamel sealing ability of the three adhesive materials in the fluorosed teeth. 
